# Secondary Degeneration Impairs Myelin Ultrastructural Development in Adulthood following Adolescent Neurotrauma in the Rat Optic Nerve

**DOI:** 10.3390/ijms24043343

**Published:** 2023-02-07

**Authors:** Brittney R. Lins, Chidozie C. Anyaegbu, Terence McGonigle, Sarah C. Hellewell, Parth Patel, Harry Reagan, Cara Rooke-Wiesner, Andrew Warnock, Michael Archer, Jan M. Hemmi, Carole Bartlett, Melinda Fitzgerald

**Affiliations:** 1 Curtin Health Innovation Research Institute, Curtin University, Bentley, WA 6845, Australia; 2 Perron Institute for Neurological and Translational Sciences, Nedlands, WA 6009, Australia; 3 School of Biological Sciences, The University of Western Australia, Perth, WA 6009, Australia; 4 Oceans Institute, The University of Western Australia, Perth, WA 6009, Australia

**Keywords:** myelin, axon, white matter, neurodevelopment, CNS injury, oligodendrocyte, transmission electron microscopy, ultrastructure

## Abstract

Adolescence is a critical period of postnatal development characterized by social, emotional, and cognitive changes. These changes are increasingly understood to depend on white matter development. White matter is highly vulnerable to the effects of injury, including secondary degeneration in regions adjacent to the primary injury site which alters the myelin ultrastructure. However, the impact of such alterations on adolescent white matter maturation is yet to be investigated. To address this, female piebald-virol-glaxo rats underwent partial transection of the optic nerve during early adolescence (postnatal day (PND) 56) with tissue collection two weeks (PND 70) or three months later (PND 140). Axons and myelin in the transmission electron micrographs of tissue adjacent to the injury were classified and measured based on the appearance of the myelin laminae. Injury in adolescence impaired the myelin structure in adulthood, resulting in a lower percentage of axons with compact myelin and a higher percentage of axons with severe myelin decompaction. Myelin thickness did not increase as expected into adulthood after injury and the relationship between the axon diameter and myelin thickness in adulthood was altered. Notably, dysmyelination was not observed 2 weeks postinjury. In conclusion, injury in adolescence altered the developmental trajectory, resulting in impaired myelin maturation when assessed at the ultrastructural level in adulthood.

## 1. Introduction

The central nervous system (CNS) develops and matures throughout childhood, adolescence, and into adulthood, with changes to myelination being a key component of development. In the human CNS, global white matter content displays a generally linear increase with age that persists to at least age 20, though several studies report increases into the fourth decade [1,2,3,4]. Adolescence is a critical period of postnatal neurodevelopment characterized by rapidly developing social, emotional, and cognitive abilities. Healthy myelin maturation underlies brain development and is characterized by overall increased myelin content in both grey and white matter; higher volume of white matter; and higher fractional anisotropy which suggests increased structural integrity of myelinated tracts [3,5,6,7]. These changes are associated with the development of thicker myelin, increased axon diameter, and increased density and organization of white matter tracts [6,8,9]. In older adolescents and young adults, these changes are seen mainly in frontal connections, aligning with the development of complex cognition and inhibition that occurs at this age [4,10]. An altered trajectory of adolescent myelin maturation is increasingly associated with the emergence of mental health disorders such as generalized anxiety disorder, mood disorders, and psychiatric illnesses [11,12,13,14,15], and understanding the factors that disrupt this maturation process may improve the understanding of these disorders. Impaired myelin maturation is seen in both humans and animals after psychological and physical trauma in early life, such as infant neglect, stress, socioeconomic disadvantage, and traumatic brain injury [11,16,17,18], demonstrating myelin and white matter vulnerability to developmental disruption.

CNS injury is common, can occur throughout life, and is associated with myelin and white matter disruption [18,19]. Therefore, CNS injury is a factor of interest in the trajectory of white matter maturation, as well as overall myelin health and function. In addition to the immediate physical damage induced at the focal injury site, CNS injury triggers subsequent events that affect surrounding tissue areas and induce downstream pathological consequences that result in the damage of otherwise uninjured cells in a process known as secondary degeneration [20,21,22]. Secondary degeneration is self-propagating and characterized by the release of calcium (Ca^2+^) stores, proteases, excitotoxic glutamate, calcium-induced calcium release, and the production of reactive oxygen and nitrogen species, which lead to oxidative damage [23,24].

Myelin-producing oligodendrocytes are highly susceptible to the secondary effects of injury [25,26]. Oligodendrocytes are more vulnerable to unsequestered Ca^2+^ influx than other CNS cells due to the higher expression of cation channels, such as adenosine triphosphate receptor P2X_7_ and α-amino-3-hydroxy-5-methyl-4-isoxazolepropionic acid (AMPA) glutamate receptors [27,28]. AMPA receptor-mediated desensitization, a feedback mechanism that limits AMPA receptor permeability when overly stimulated, is lower in oligodendrocytes and oligodendrocyte progenitor cells [29]. Low levels of endogenous antioxidant defenses and high levels of redox-active iron increase vulnerability to reactive species, and their high lipid content is susceptible to lipid peroxidation [30,31,32]. This enhanced vulnerability to secondary degeneration may not only underlie the progressive nature of CNS injury, but also increase susceptibility to a disrupted trajectory of white matter development when injury occurs around a developmental window such as adolescence. Improved understanding of these processes may lead to strategies for preventing secondary degeneration and promoting healthy CNS maturation following adverse events.

**Figure 1:**
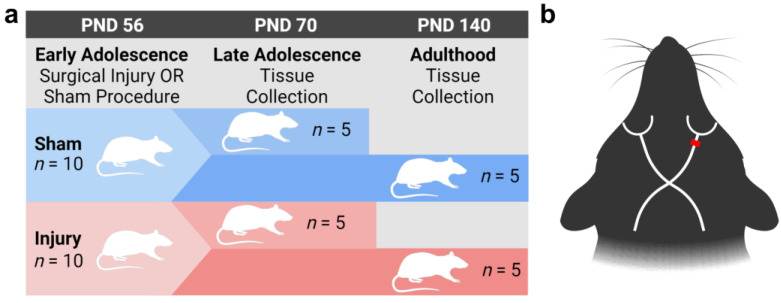
Experimental schematics. Experimental timeline of surgical injury via partial optic nerve transection, or sham procedure, on PND 56 (early adolescence) relative to tissue collection either 2 weeks later on PND 70 (late adolescence) or 3 months later on PND 140 (adulthood) (**a**). Schematic of rat head with superimposed optic nerve tracts. The red dash represents the location of partial optic nerve transection, or sham procedure, 1 mm behind the right eye (**b**).

Through high-resolution transmission electron microscopy studies, we and others have demonstrated ultrastructural changes to white matter tracts after CNS injury [20,21,33,34,35,36,37,38,39,40,41,42,43]. These alterations include disrupted myelin structure, such as decompaction of the myelin lamellae, and thinner myelin relative to the axon diameter [34,42]. Axonal changes include the loss of axons and axonal swelling, with these changes occurring progressively and persisting for at least 6 months [34]. To our knowledge, these effects are yet to be assessed in a developmental context. In this study, we modelled CNS injury in adolescence using surgical partial transection of the optic nerve in rats, where the primary injury site on the dorsal aspect can be readily distinguished from the adjacent ventral region, which is vulnerable to secondary degeneration [20,21]. Optic nerve tissue was collected 2 weeks or 3 months later, alongside age-matched sham-injured groups to investigate the axon and myelin ultrastructural changes in development, with and without CNS injury in adolescence (Figure 1).

## 2. Results

### 2.1. Injury in Adolescence Was Associated with a Lower Percentage of Axons with Compact Myelin and a Higher Percentage of Axons with Severely Decompacted Myelin at Adulthood

**Figure 2:**
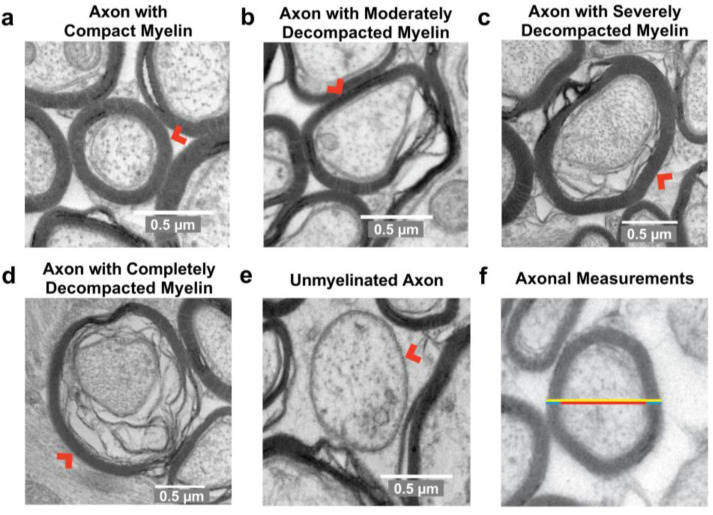
Representative images of axon cross-sections classified based on myelin compaction. Axons with compact myelin were characterized by thick, high electron density (dark appearance) myelin with <10% axon circumference affected by decompaction (**a**). Axons with moderately decompacted myelin had 10–35% of the axon circumference affected by decompaction (**b**). Axons with severely decompacted myelin had 35–99% of the axon circumference affected by decompaction (**c**). Axons with completely decompacted myelin were characterized by decompacted myelin surrounding the entire axon circumference (**d**). Unmyelinated axons had a round or oval appearance with no surrounding myelin (**e**). Depiction of axonal morphology measurements. Axon diameter was measured at the minimum distance (red); myelin thickness was measured at the same location, parallel to the diameter measurement and presented as an average of the 2 sides (blue); fiber diameter was measured at the same location, parallel to axon diameter and included myelin, axon, and axolemmal space if present (yellow) (**f**). Red arrows indicate representative axon. Scale bars = 0.5 µm.

**Figure 3:**
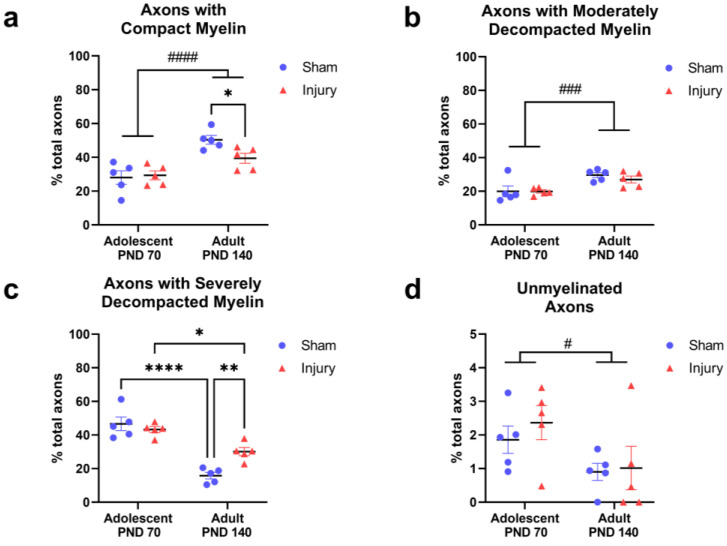
Percentage of axons classified based on myelin appearance in sham and injured rats at adolescence (PND 70) and adulthood (PND 140). Data were averaged per animal and presented as group (*n* = 5) means ± SEM for the following axon classifications: axons with compact, electron-dense myelin (**a**); axons with moderate myelin decompaction (**b**); axons with severely decompacted myelin (**c**); unmyelinated axons (**d**). Statistical comparisons were made between groups. #### indicate main effect *p* < 0.0001; ### *p* < 0.001; # *p* < 0.05. **** indicate post hoc comparison *p* < 0.0001; ** *p* < 0.01; * *p* < 0.05. Additional significant effects were omitted from panel (**c**) for space and clarity, including a main effect of age (*p* < 0.0001).

Axons were classified based on the appearance of their ensheathing myelin (Figure 2) and quantified as a percentage of the total axons classified (Figure 3). Between adolescence and adulthood, the percentage of axons with compact myelin increased overall (F_(1,16)_ = 27.36, *p* < 0.0001; Figure 3a) and the interaction between injury and age did not achieve statistical significance (F_(1,16)_ = 3.912, *p* = 0.0654). Post hoc analysis revealed a significantly greater percentage of axons with compact myelin in the sham-adult group relative to the injury-adult group (*p* = 0.0482) while the adolescent groups were not different. The percentage of axons with moderately decompacted myelin also increased with age (F_(1,16)_ = 16.20, *p* = 0.0010; Figure 3b).

In accordance with the increased amount of compact and moderately decompacted myelin with age, the percentage of axons with severely decompacted myelin decreased with age (F_(1,16)_ = 66.91, *p* < 0.0001; Figure 3c). A significant interaction between age and injury (F_(1,16)_ = 10.80, *p* = 0.0046) prompted post hoc comparison of all groups. The percentage of axons with severely decompacted myelin decreased with age in both the sham (*p* < 0.0001) and injury (*p* = 0.0153) groups, and the sham-adult rats had a further significant decrease relative to the injury-adult rats (*p* = 0.0084). Taken together, myelin became more compact with maturation, moving from higher proportions of axons with severely decompacted myelin toward axons with more compact myelin, with a greater magnitude of change observed in the sham groups relative to the injury groups (Figure 3a–c). The proportion of unmyelinated axons also decreased with age (F_(1,16)_ = 5.871, *p* = 0.0276; Figure 3d), further indicating a maturation effect.

Axons with additional myelin classifications were analyzed, including lightly myelinated axons, axons with complete myelin decompaction, and axons with paranodal profiles. Neither age nor injury significantly affected these axonal populations, and they represented a small proportion of the total axons analyzed.

### 2.2. Injury in Adolescence Prevented Development of Thicker, more Compact Myelin in Adulthood

**Figure 4:**
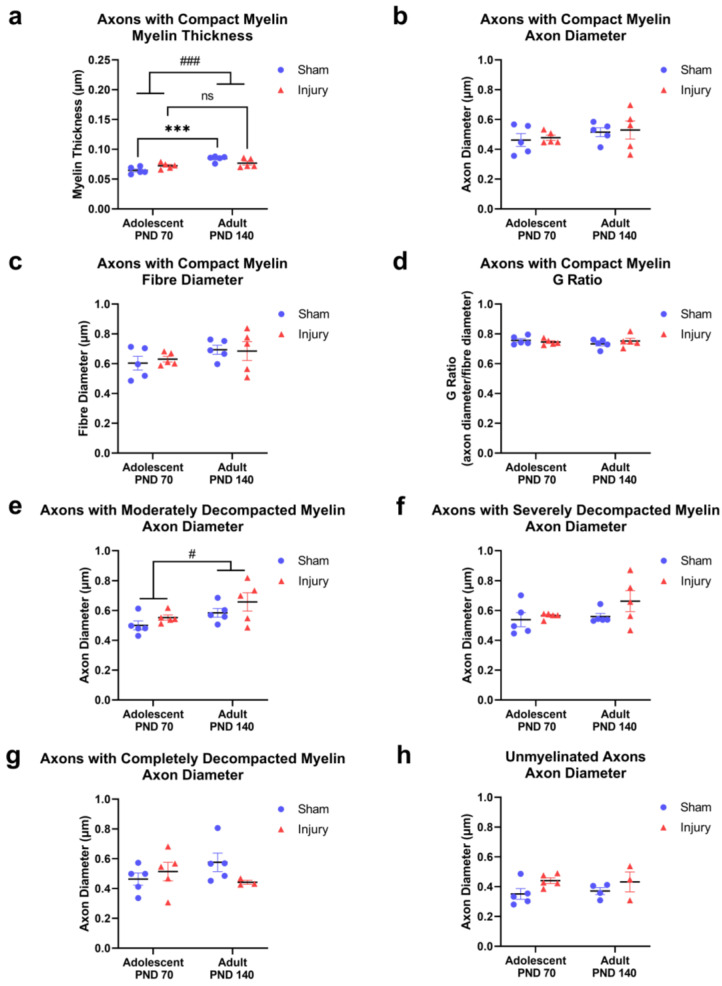
Morphology measurements of axons with compact myelin and diameter measurements of axons with decompacted myelin in sham and injured rats at adolescence (PND 70) and adulthood (PND 140)**.** In axons with compact myelin, the following morphological features were quantified by length (µm) then averaged at the level of animal and compared across groups (*n* = 5): myelin thickness (**a**); axon diameter (**b**); fiber diameter (**c**); G ratio (**d**). In axons with myelin decompaction or no myelin, axon diameter was quantified by length (µm) then averaged at the level of the animal and compared across groups (*n* = 5, but *n* = 4 and *n* = 3 in panel h for PND 140 groups) and displayed for the following axon classifications: axons with moderately decompacted myelin (**e**); axons with severely decompacted myelin (**f**); axons with completely decompacted myelin (**g**); unmyelinated axons (**h**). Data were presented as means ± SEM. Statistical comparisons were made between groups. ### indicate main effect *p* < 0.001; # *p* < 0.05. *** indicate post hoc comparison *p* < 0.001. ns indicate not significant.

In the axons with compact myelin, the myelin thickness significantly increased with age (F_(1,16)_ = 21.05, *p* = 0.0003; Figure 4a). A significant interaction revealed that maturation affects the myelin thickness differently in the injury and sham groups (F_(1,16)_ = 8.525, *p* = 0.0100, Figure 4a). Post hoc comparison indicated only the sham groups displayed an increased myelin thickness with maturation (*p* = 0.0001) while the myelin thickness in the injury groups remained similar to that observed in adolescence and did not change in adulthood (*p* = 0.5107). Additional morphological features of axons with compact myelin were not significantly altered with age or injury (Figure 4b–d; Supplementary Table S1).

### 2.3. The Diameter of Axons with Moderate Myelin Decompaction Increased with Age, but Not Injury

Axons with moderate myelin decompaction increased in diameter between adolescence and adulthood (F_(1,16)_ = 6.096, *p* = 0.0252; Figure 4e). The diameter of the axons with more severe myelin decompaction did not change as a result of maturation or injury (Figure 4e–g; Supplementary Table S1). The effect of injury on the diameter of the unmyelinated axons did not reach statistical significance (F_(1,13)_ = 4.47, *p* = 0.0542, Figure 4h). It should be noted that unmyelinated axons were not observed in three animals (one in the sham-adult group and two in the injury-adult group) which resulted in a reduced *n* = 4 and *n* = 3, respectively, and likely reduced the power of this analysis.

### 2.4. The Relationship between Axon Diameter and Myelin Thickness Deteriorated in Adulthood after Injury in Early Adolescence

**Figure 5:**
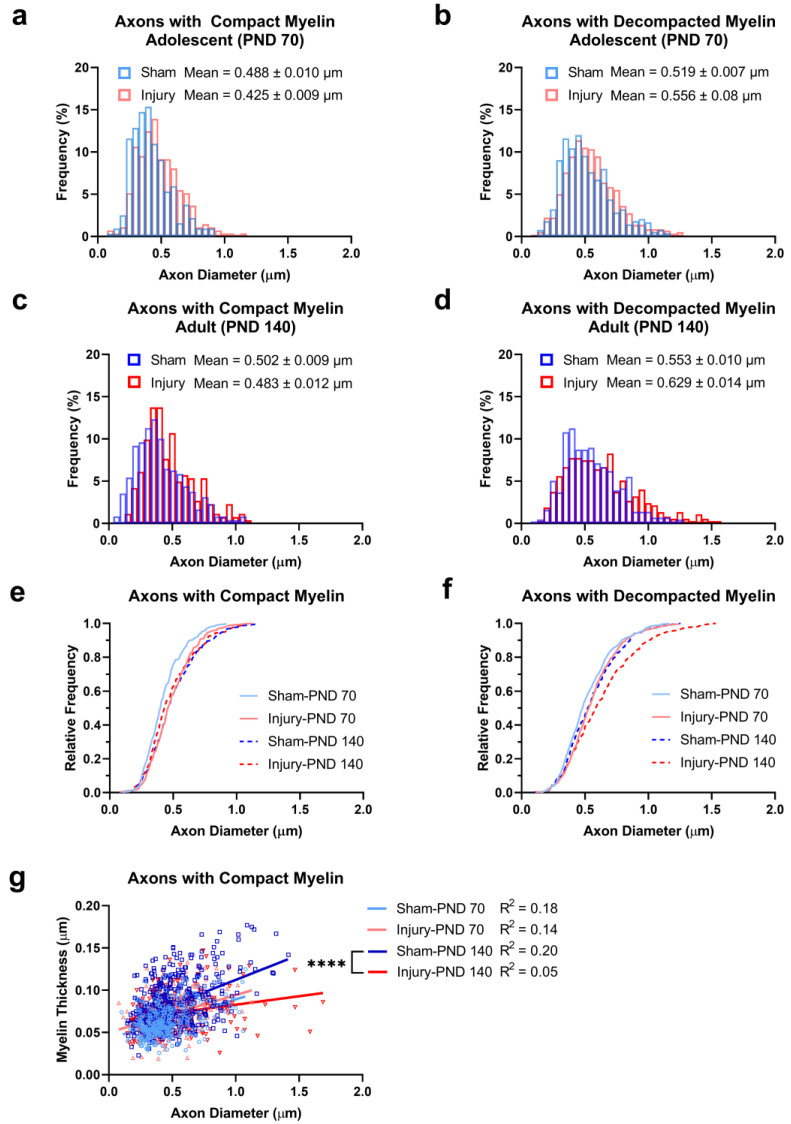
Population analyses of axon diameter and myelin thickness distributions. Overlaid histogram plots display the frequency distributions of all axons with compact myelin in injury- and sham-adolescent (PND 70) rats (**a**), and similarly for axons with decompacted myelin (representing all axons with 10–100% circumference affected by decompaction) in adolescent (PND 70) rats (**b**). Overlaid histogram plots displayed the frequency distributions of all axons with compact myelin in injury- and sham-adult (PND 140) rats (**c**), and similarly for all axons with decompacted myelin in adult (PND 140) rats (**d**). Cumulative frequency plots illustrate the distribution of axon diameters in all axons with compact myelin (**e**) and all axons with decompacted myelin (**f**) at the population level. A scatter plot including all data illustrated the relationship between myelin thickness and axon diameter in axons with compact myelin with overlaid lines of best fit (**g**). **** indicate slope comparison *p* < 0.0001.

Histograms were created to display the distribution of the axon diameter across the entire population of the axons measured. These were grouped by myelin characterization as either compact (<10% circumference decompacted) or decompacted (10–100% circumference decompacted; Figure 5a–d). A cumulative frequency plot of the diameter of the axons with compact myelin revealed a shift to smaller diameters (left shift) in the adolescent-sham group population relative to the other population curves (Figure 5e). In addition, the cumulative frequency plot of the axon diameter in the decompacted myelin category indicated an apparent shift to larger diameters (right shift) in the injury-adult population relative to all other groups (Figure 5f). To statistically compare these populations and control for pseudoreplication, the median diameter of the axons with compact myelin and median diameter of the axons with decompacted myelin were determined for each individual rat (*n* = 20) and ranked from 1–20. The ranks were then grouped by injury and age and compared using 2-way ANOVA. However, no significant differences between the median axon diameter were observed in the axons with compact myelin (Figure 5e; Supplementary Table S1) or decompacted myelin (Figure 5f; Supplementary Table S1).

The relationship between the myelin thickness and axon diameter in the axons with compact myelin was determined using linear regression (Figure 5g). The R^2^ values from the sham and injured groups at adolescence were comparable (sham-adolescence: R^2^ = 0.18; injury-adolescence: R^2^ = 0.14); however, these values diverged between the sham and injury groups at adulthood (sham-adult: R^2^ = 0.20; injury-adult: R^2^ = 0.05), indicating that the axon diameter was a poor predictor of the myelin thickness in adulthood when CNS injury occurred in adolescence. The slopes of the lines of best fit were compared in a pairwise fashion between the sham and injury groups of the same age. In adolescence, the slopes were not significantly different (F_(1,587)_ = 0.071, *p* = 0.7904; Figure 5g); however, significant differences between the slopes emerged at adulthood (F_(1,739)_ = 24.72, *p* < 0.0001; Figure 5g). A secondary analysis was conducted to determine whether this effect was driven by the largest diameter axons; however, with the 15 largest axons removed from the analysis (*n* = 7 from the injury-adult group and *n* = 8 from the sham-adult group), the slopes remained significantly different at adulthood (F_(1,724)_ = 10.26, *p* = 0.0014).

## 3. Discussion

We used partial transection of the optic nerve to model CNS injury in early adolescence to determine whether impaired myelin maturation is a feature of secondary degeneration after injury. We found that injury in adolescence was associated with impaired myelin maturation, assessed at the ultrastructural level at adulthood. Adult rats that experienced injury early in adolescence had a higher percentage of axons with severely decompacted myelin; a lower percentage of axons with compact myelin; and did not develop thicker myelin when compared to age-matched sham-injured controls (see results summary in Table 1).

### 3.1. Injury in Adolescence Impaired Myelin Maturation

Myelin maturation is a key component of healthy neurodevelopment characterized by the improved structural integrity of white matter tracts [44]. Maturational changes to the myelin thickness and compact structure were impaired at adulthood after injury in adolescence. These ultrastructural abnormalities may disrupt axonal function as both the myelin thickness and structure are known contributors to signal transduction velocity, and decompacted myelin results in disrupted distribution of voltage-gated sodium channels along the axon and reduced action potential velocity [45,46,47]. In the mouse optic nerve, maturational changes such as increased myelin thickness and axon diameter occur to at least PND 56 and are associated with the increased expression of sodium channel Nav 1.6 at the node of Ranvier. Together, the myelin thickness, axon diameter, and Nav 1.6 expression had a greater impact on increasing the signal transduction speed than changes to the G ratio in mice, and changes that supported faster conductance were associated with increasing age [48]. Although inter-species variation may exist between mice and rats, our data in rats suggest that the myelin thickness and compactness continued to increase between PND 70 and PND 140 in the uninjured optic nerve, which likely reflects improved conduction. Consequently, delayed or impaired myelin maturation, characterized by relatively thinner myelin and higher levels of severe decompaction as observed in the injury-adult group, is likely associated with the reduced action potential speed and signal conductance velocity. Indeed, the functional effects of secondary degeneration in the optic nerve model employed in the present study include impaired visual function in the optokinetic nystagmus test in adult animals [35,38]. In human childhood and adolescence, the functional significance of white matter maturation includes a faster processing speed which underlies the development of complex reasoning skills [49]. White matter integrity continues to support fast processing speed and cognitive abilities in adulthood [50], so impaired myelin maturation as a result of secondary degeneration, such as seen in our rat model, may have long-lasting functional consequences. The application of these findings between rodents and humans must consider differences between species, not limited to the developmental time scale, developmental age at birth, and gyrencephalic or lissencephalic brain structure [1]. While the timescale of human development is not perfectly recapitulated in rodents, mammalian neurodevelopment is highly conserved, and rodents are generally considered a good model system for human neurodevelopment [1].

### 3.2. Ultrastructure of Myelin Matured between Adolescence and Adulthood

The axon and myelin ultrastructure in the optic nerve changed between adolescence and adulthood in female rats, with the greatest changes observed in the sham-injury groups. Specifically, there was a general progression from axons with severely decompacted myelin in adolescence to axons with more compact myelin at adulthood, alongside a thicker compact myelin and reduced percentage of unmyelinated axons. These changes are consistent with healthy neurodevelopment, which is well established to occur in both animal models and through human neuroimaging studies [1,2,5,11,17,51]. Diffusion-weighted imaging is commonly used to measure the structural integrity of white matter tracts in vivo, with fractional anisotropy a commonly reported metric for white matter integrity. Increasing fractional anisotropy over the course of adolescence is well established in humans, with these changes attributed to the microstructural alterations of: increased myelin thickness; the packing of axons into denser tracts; increasing axon diameter; and changes to the internal axonal structure [9,52,53,54]. These findings align with our observed increase in the myelin thickness around axons with compact myelin; increased proportions of axons with compact myelin; and reduced proportions of unmyelinated axons in adulthood relative to adolescence.

Interestingly, the only axon population with an increased axon diameter at adulthood was the axons with moderately decompacted myelin, regardless of the injury or sham group. This finding was somewhat unexpected, as we have reported an increased axonal diameter with injury in axons with compact myelin in this model [33,34]. This finding may reflect the younger age of injury, more complex statistical analysis of our two-way design, and/or it is possible that maturation changes to the axon diameter are specific to axons where myelin is moderately decompacted. In rats, the most rapid rates of axon caliber change occur in the distal optic nerve between PND 12–16 and 21–30, with a continued but gradual increase reported to PND 120 [51]. Our time points (PND 56–140) and imaging location (proximal to retina) may not have captured a developmental period of significant increase to the axon caliber. Axon–oligodendrocyte interactions provide extrinsic signals that increase the axon caliber during postnatal development regardless of myelination status [51], so it can be inferred from our data that the degree of myelin compaction may also influence changes to the axon diameter in the transition from adolescence to adulthood in rat.

### 3.3. Overt Myelin Ultrastructural Changes Were Not Observed 2 Weeks after Injury

The absence of overt ultrastructural dysmyelination 2 weeks after injury contributes to understanding the progressive nature of secondary degeneration. Signs of inflammation and oxidative stress are evident in regions adjacent to injury within minutes of partial nerve transection, and myelin pathology such as increased paranodal gap length and increased percentage of abnormal node–paranode complexes emerge by the following day [37,39,55]. However, the effects of secondary degeneration on myelin over time are dynamic. Node–paranode abnormalities are present 3 days after injury but resolve at 1 week, only to re-emerge 1 month after injury and persist chronically to at least 3 months [37]. The first week after partial transection is characterized by the increased proliferation of oligodendrocyte progenitor cells (OPCs), though this population remains chronically depleted 1 and 3 months later in areas vulnerable to secondary degeneration. OPCs and mature myelinating oligodendrocytes vulnerable to secondary degeneration display oxidative damage to DNA at 3 days after injury, with oligodendrocytes derived after injury less likely to die [32]. Notably, oligodendrocytes derived after injury display reduced myelin regulatory factor mRNA by 28 days after injury, suggesting an impaired myelinating capacity [32]. Together, the depleted OPC population, loss of mature oligodendrocytes, and potentially impaired myelination capacity of postinjury-derived oligodendrocytes may contribute to chronic myelin ultrastructure abnormalities after injury in adult animals [32,33,34,36,37]. We did not see overt ultrastuctural dysmyelination 2 weeks after injury in the present study, but myelin pathology emerged in the intervening three-month period. This aligns with previous findings that acute pathology resolves within a week of partial transection injury, yet it appears axons and myelin remain vulnerable to chronic abnormalities in both a developmental context and following injury in adulthood. Support to the axon before the emergence of chronic dysmyelination may be worthy of exploration from a therapeutic standpoint. Therapeutic agents that improve the myelin structure and tract function in secondary degeneration have been identified [20,38,56,57,58,59,60,61], and it is possible that employment of these strategies during adolescence may prevent white matter maturation deficits following CNS injury, as well as prevent functional loss after injury in general.

## 4. Materials and Methods

### 4.1. Animals

Female piebald-virol-glaxo (PVG) rats (*n* = 20; 140–166 g; Animal Resource Centre, Murdoch, WA, Australia) arrived at postnatal day (PND) 49 and acclimated for 7 days prior to commencing experimental procedures. Animals were housed 2–3 to a cage in standard housing within a temperature-controlled environment (21 °C) on an automatic light–dark cycle (12 h:12 h) with food and water available ad libitum. In a 2 × 2 factorial research design, rats were randomly allocated to undergo either partial transection injury or sham surgical procedures on approximately PND 56 (early adolescence) [62], and for tissue collection either 2 weeks (approximately PND 70, late adolescence) or 3 months (approximately PND 140, adulthood) later. Except for routine postoperative monitoring and husbandry, rats were undisturbed until humane euthanasia. Groups (*n* = 5) were designated as sham-adolescent, injury-adolescent, sham-adult, or injury-adult (Figure 1a). Note that n of 3–8 is standard in electron microscopy studies where high granularity assessments of multiple measures are conducted [33,34,35,37,38,40,41,63,64,65,66].

### 4.2. Optic Nerve Partial Transection Surgery

Surgical partial transection of the optic nerve was conducted as previously described [20,21]. Rats were deeply anesthetized using a combination of xylazine (10 mg/kg, intraperitoneal (i.p.)) and ketamine (50 mg/kg, i.p.). The right optic nerve was exposed, and an incision made 1 mm behind the eye in the dorsal aspect of the exposed optic nerve to a depth of 200 µm with a diamond radial keratotomy knife (Figure 1b). Following partial transection, tissue was positioned to approximate its natural position and the opening incision sutured. Postsurgical recovery included monitoring on a warming blanket, and antibiotic and analgesic treatments (Neomycin, 10 mg/kg in sterile PBS subcutaneous (s.c.); Norocarp, 2.8 mg/kg, s.c; Tricin, Jurox, Rutherford, Australia). Sham groups underwent identical surgical procedures with exclusion of the optic nerve incision.

### 4.3. Tissue Preparation

Optic nerve tissue was collected and processed in accordance with previously described protocols [20,33,34,67]. Two weeks or three months following injury or sham procedures, rats were euthanized with Euthal (Pentobarbitone sodium, 850 mg/kg; Phenytoin sodium, 125 mg/kg; i.p.) and transcardially perfused with 0.9% saline, then 2% paraformaldehyde/2.5% glutaraldehyde/2% sucrose in 0.1M phosphate buffer (pH 7.2). Dissected nerves were stored in 0.13M Sorenson’s phosphate buffer (pH 7.2). Optic nerves were further cleaned under a dissection microscope to remove excess tissue and the dura sheath, taking care to avoid distortion or stretching of the nerve.

Cleaned and trimmed optic nerves were postfixed in 1% osmium (Electron Microscopy Sciences, ProSciTech, Townsville, QLD, Australia: Cat#C011). A Lynx processor was used to dehydrate the tissue through an ethanol series to propylene oxide and tissue was then infiltrated with resin into Araldite Procure mixture (ProSciTech, Townsville, Queensland, Australia: Cat# 039). Epoxy resin-embedded tissue segments were cured for 24 h at 60 °C and serially sectioned on an ultramicrotome (LKB Nova, Bromma, Sweden). One µm transverse sections were deplasticized with saturated NaOH in 70% (*v*/*v*) ethanol and stained for 15–30 s at 95 °C in aqueous toluidine blue in 1% borax. The transverse nature of the sections was confirmed by the circular appearance of the axons. Low-power micrographs of entire sections were taken at 20× magnification to identify the injury site along the optic nerve for transmission electron microscopy (TEM) analysis. Transverse ultra-thin sections (100 nm) of optic nerve at the injury site were then cut using a diamond knife, mounted onto copper support grids (3.05 mm), and poststained with uranyl acetate and lead citrate [33,67].

### 4.4. Image Acquisition

Images of ultra-thin sections were acquired using a JEOL (2100 TEM; Tokyo, Japan). Images were visualized using Olympus iTEM Soft Imaging Solution Software and captured using an Olympus Megaview III digital camera (Oris; Gatan, Pleasanton, CA, USA). A copper grid was used to define consistent regions of sampling across sections from the ventral region of the nerve. Images were captured at a primary magnification of 4000×.

### 4.5. Ultrastructural Analysis of Myelinated Axons

All axons with the minimum axon diameter visible within the field of view of each image were analyzed. Image size was selected to permit accurate classification of myelin appearance (compact or decompacted laminae) and as a result, larger diameter axons may have been disproportionately excluded from analysis due to their higher likelihood of falling partially outside of the field of view. Axons were classified based on the profile of their myelin sheath; specifically, the extent to which the ensheathing myelin was held in compact layers, or whether separation of the myelin laminae was visible. Classifications were adapted from previous studies and defined as follows: axons with compact myelin were characterized by thick, dark myelin appearance with <10% axon circumference affected by decompaction (Figure 2a); axons with moderately decompacted myelin had 10–35% of the axon circumference affected by decompaction (Figure 2b); axons with severely decompacted myelin had 36–99% of the axon circumference with decompaction (Figure 2c); axons with completely decompacted myelin were fully ensheathed in myelin with the appearance of multiple diffuse layers, encompassing 100% of the axon diameter (Figure 2d); and unmyelinated axons with a regular round or oval appearance without surrounding myelin (Figure 2e) but distinct from glial cell processes, which are elongated in shape. Additional categories were included to describe other observed anatomical variations: paranodal profiles defined as axons with a large axolemmal space; lightly myelinated axons surrounded by thick, compact, low electron density (light-appearing) myelin with <10% axon circumference with observed decompaction; and aberrant profiles with constricted axoplasm showing empty membrane folds and aberrant extracellular compartments [33,34,63,67].

Axons characterized with ‘compact myelin’ were further analyzed to determine axon diameter, myelin thickness, fiber diameter, and G ratio (axon diameter/fiber diameter). The minimum axon diameter was used, and myelin thickness was measured at that same location (Figure 2f). The reported myelin thickness was the average of the two measurements on each side of the minimum axon diameter. Fiber diameter included the minimum axon diameter plus the myelin sheath and axolemmal space (Figure 2f). These measurements were not collected from axons with decompacted myelin because decompaction distorts the apparent thickness of myelin and fiber diameter and prevents consistent measurement. All image analyses were conducted using FIJI Image J (version 1.53) software with investigators blinded to animal identity and treatment status.

### 4.6. Statistical Analyses and Data Presentation

Data were compiled in Microsoft Excel and analyzed using GraphPad Prism (version 9.4.1, GraphPad Software, LLC, San Diego, CA, USA). Measurements were averaged at the individual animal level, and groups were compared using 2-way ANOVA. Where indicated, post hoc comparisons were conducted using Bonferroni’s multiple comparison’s test (comparing means within factors) or Tukey’s HSD test (comparing all means), based on the outcomes of the 2-way ANOVA and a priori selection of relevant comparisons of interest [68]. Cumulative frequency plots were generated to display the distribution of all collected data points. To avoid pseudoreplication, comparisons of these populations were conducted by calculating the median for each individual animal and conducting 2-way ANOVA on median rank. Distribution of the residuals was normal (Shapiro–Wilk). To gain accurate visual representation of the data, scatter plots of axon diameter and myelin thickness included all collected data points while linear regression analyses were conducted in a pairwise fashion between injury and sham groups. No animals were excluded from analysis. In the population histograms, cumulative frequency plots, and linear regression analysis, outliers among the individual data points were identified using the ROUT method in GraphPad Prism and these were removed from graphing and analysis. Graphs were made using GraphPad Prism. All data are presented as averages ± SEM, except the population analyses (Figure 5) which depict all measurements.

## 5. Conclusions

Maturation of the myelin ultrastructure was impaired in the rat optic nerve at adulthood following injury in early adolescence. Maturation observed in the sham-injured controls included thicker myelin with age; increased percentage of axons with compact myelin; decreased percentage of axons with severely decompacted myelin; and decreased percentage of unmyelinated axons, but injury prevented or reduced the magnitude of these changes. Overt dysmyelination was not observed 2 weeks after PT injury, suggesting a window of opportunity for therapeutic intervention where the axon and myelin structure remains intact, but vulnerable to the emergence of chronic pathology.

## Figures and Tables

**Table 1:** Summary of maturation changes to myelin and axons between PND 70-140, with and without injury.

	**Adult** **(PND 140)** **Relative to Adolescent** **(PND 70)** **Overall**	**Injury-Adolescent** **(PND 70)** **Relative to** **Sham-Adolescent** **(PND 70)**	**Sham-Adult** **(PND 140)** **Relative to** **Sham-Adolescent (PND 70)**	**Injury-Adult (PND 140)** **Relative to** **Sham-Adult** **(PND 140)**	**Injury-Adult (PND 140)** **Relative to** **Injury-Adolescent (PND 70)**
**Myelin Classifications**					
Axons with Compact Myelin (%)	↑↑	ns	―	↓	―
Axons with moderately decompacted myelin (%)	↑↑	―	―	―	―
Axons with severely decompacted myelin (%)	↓↓	ns	↓↓	↑	↓
Unmyelinated axons (%)	↓	―	―	―	―
**Morphology of Axons with Compact Myelin**					
Myelin Thickness (µm)	↑↑	―	↑↑	―	ns
Axon Diameter (µm)	ns	―	―	―	―
Fibre Diameter (µm)	ns	―	―	―	―
G Ratio	ns	―	―	―	―
**Diameter of Axons with Decompacted Myelin**					
Axons with Moderately Decompacted Myelin (µm)	↑	―	―	―	―
Axons with Severely Decompacted Myelin (µm)	ns	―	―	―	―
Axons with Completely Decompacted Myelin (µm)	ns	―	―	―	―
Unmyelinated Axons (µm)	ns	―	―	―	―

Arrows ↑ and ↓ indicate a statistically significant increase or decrease, respectively; single arrows indicate *p* > 0.001 and <0.05; double arrows indicate *p* ≤  0.001; ns indicate not significant; ― indicate not compared.

## Data Availability

Data available upon request.

## Supplementary Materials

The following supporting information can be downloaded at: https://www.mdpi.com/article/10.3390/ijms24043343/s1.

## References

[1] Semple B.D., Blomgren K., Gimlin K., Ferriero D.M., Noble-Haeusslein L.J. (2013). Brain development in rodents and humans: Identifying benchmarks of maturation and vulnerability to injury across species. Prog. Neurobiol..

[2] Giedd J.N., Blumenthal J., Jeffries N.O., Castellanos F.X., Liu H., Zijdenbos A., Paus T., Evans A.C., Rapoport J.L. (1999). Brain development during childhood and adolescence: A longitudinal MRI study. Nat. Neurosci..

[3] Lebel C., Beaulieu C. (2011). Longitudinal Development of Human Brain Wiring Continues from Childhood into Adulthood. J. Neurosci..

[4] Lebel C., Gee M., Camicioli R., Wieler M., Martin W., Beaulieu C. (2012). Diffusion tensor imaging of white matter tract evolution over the lifespan. NeuroImage.

[5] Corrigan N.M., Yarnykh V.L., Hippe D.S., Owen J.P., Huber E., Zhao T.C., Kuhl P.K. (2021). Myelin development in cerebral gray and white matter during adolescence and late childhood. NeuroImage.

[6] Barnea-Goraly N., Menon V., Eckert M., Tamm L., Bammer R., Karchemskiy A., Dant C.C., Reiss A.L. (2005). White matter development during childhood and adolescence: A cross-sectional diffusion tensor imaging study. Cereb. Cortex.

[7] Ashtari M., Cervellione K.L., Hasan K.M., Wu J., McIlree C., Kester H., Ardekani B.A., Roofeh D., Szeszko P.R., Kumra S. (2007). White matter development during late adolescence in healthy males: A cross-sectional diffusion tensor imaging study. NeuroImage.

[8] Giorgio A., Watkins K.E., Chadwick M., James S., Winmill L., Douaud G., De Stefano N., Matthews P.M., Smith S.M., Johansen-Berg H. (2010). Longitudinal changes in grey and white matter during adolescence. NeuroImage.

[9] Beaulieu C. (2002). The basis of anisotropic water diffusion in the nervous system—A technical review. NMR Biomed..

[10] Sowell E.R., Thompson P.M., Holmes C.J., Jernigan T.L., Toga A.W. (1999). In vivo evidence for post-adolescent brain maturation in frontal and striatal regions. Nat. Neurosci..

[11] Vanes L.D., Moutoussis M., Ziegler G., Goodyer I.M., Fonagy P., Jones P.B., Bullmore E.T., Consortium N., Dolan R.J. (2020). White matter tract myelin maturation and its association with general psychopathology in adolescence and early adulthood. Hum. Brain Mapp..

[12] Paus T., Keshavan M., Giedd J.N. (2008). Why do many psychiatric disorders emerge during adolescence?. Nat. Rev. Neurosci..

[13] Romero S., de la Serna E., Baeza I., Valli I., Pariente J.C., Picado M., Bargalló N., Sugranyes G., Castro-Fornieles J. (2022). Altered White Matter Integrity at Illness Onset in Adolescents With a First Episode of Psychosis. Front. Psychiatry.

[14] Heng S., Song A.W., Sim K. (2010). White matter abnormalities in bipolar disorder: Insights from diffusion tensor imaging studies. J. Neural. Transm..

[15] Kim M.J., Whalen P.J. (2009). The Structural Integrity of an Amygdala–Prefrontal Pathway Predicts Trait Anxiety. J. Neurosci..

[16] Ziegler G., Moutoussis M., Hauser T.U., Fearon P., Bullmore E.T., Goodyer I.M., Fonagy P., Jones P.B., Lindenberger U., Dolan R.J. (2020). Childhood socio-economic disadvantage predicts reduced myelin growth across adolescence and young adulthood. Hum. Brain Mapp..

[17] Howell B.R., McCormack K.M., Grand A.P., Sawyer N.T., Zhang X., Maestripieri D., Hu X., Sanchez M.M. (2013). Brain white matter microstructure alterations in adolescent rhesus monkeys exposed to early life stress: Associations with high cortisol during infancy. Biol. Mood Anxiety Disord..

[18] Tasker R.C. (2006). Changes in White Matter Late after Severe Traumatic Brain Injury in Childhood. Dev. Neurosci..

[19] Badhiwala J.H., Wilson J.R., Fehlings M.G. (2019). Global burden of traumatic brain and spinal cord injury. Lancet Neurol..

[20] Fitzgerald M., Bartlett C.A., Evill L., Rodger J., Harvey A.R., Dunlop S.A. (2009). Secondary degeneration of the optic nerve following partial transection: The benefits of lomerizine. Exp. Neurol..

[21] Fitzgerald M., Payne S.C., Bartlett C.A., Evill L., Harvey A.R., Dunlop S.A. (2009). Secondary retinal ganglion cell death and the neuroprotective effects of the calcium channel blocker lomerizine. Investig. Ophthalmol. Vis. Sci..

[22] Levkovitch-Verbin H., Quigley H.A., Martin K.R., Zack D.J., Pease M.E., Valenta D.F. (2003). A model to study differences between primary and secondary degeneration of retinal ganglion cells in rats by partial optic nerve transection. Investig. Ophthalmol. Vis. Sci..

[23] Staal J.A., Dickson T.C., Gasperini R., Liu Y., Foa L., Vickers J.C. (2010). Initial calcium release from intracellular stores followed by calcium dysregulation is linked to secondary axotomy following transient axonal stretch injury. J. Neurochem..

[24] Giza C.C., Hovda D.A. (2014). The new neurometabolic cascade of concussion. Neurosurgery.

[25] Baumann N., Pham-Dinh D. (2001). Biology of oligodendrocyte and myelin in the mammalian central nervous system. Physiol. Rev..

[26] Giacci M., Fitzgerald M. (2018). Oligodendroglia Are Particularly Vulnerable to Oxidative Damage After Neurotrauma In Vivo. J. Exp. Neurosci..

[27] Matute C., Torre I., Pérez-Cerdá F., Pérez-Samartín A., Alberdi E., Etxebarria E., Arranz A.M., Ravid R., Rodríguez-Antigüedad A., Sánchez-Gómez M. (2007). P2X(7) receptor blockade prevents ATP excitotoxicity in oligodendrocytes and ameliorates experimental autoimmune encephalomyelitis. J. Neurosci..

[28] Borges K., Ohlemeyer C., Trotter J., Kettenmann H. (1994). AMPA/kainate receptor activation in murine oligodendrocyte precursor cells leads to activation of a cation conductance, calcium influx and blockade of delayed rectifying K^+^ channels. Neuroscience.

[29] Yamaya Y., Yoshioka A., Saiki S., Yuki N., Hirose G., Pleasure D. (2002). Type-2 astrocyte-like cells are more resistant than oligodendrocyte-like cells against non-N-methyl-D-aspartate glutamate receptor-mediated excitotoxicity. J. Neurosci. Res..

[30] Thorburne S.K., Juurlink B.H. (1996). Low glutathione and high iron govern the susceptibility of oligodendroglial precursors to oxidative stress. J. Neurochem..

[31] Bernardo A., Bianchi D., Magnaghi V., Minghetti L. (2009). Peroxisome proliferator-activated receptor-gamma agonists promote differentiation and antioxidant defenses of oligodendrocyte progenitor cells. J. Neuropathol. Exp. Neurol..

[32] Giacci M.K., Bartlett C.A., Smith N.M., Iyer K.S., Toomey L.M., Jiang H., Guagliardo P., Kilburn M.R., Fitzgerald M. (2018). Oligodendroglia Are Particularly Vulnerable to Oxidative Damage after Neurotrauma In Vivo. J. Neurosci..

[33] Payne S.C., Bartlett C.A., Harvey A.R., Dunlop S.A., Fitzgerald M. (2011). Chronic swelling and abnormal myelination during secondary degeneration after partial injury to a central nervous system tract. J. Neurotrauma.

[34] Payne S.C., Bartlett C.A., Harvey A.R., Dunlop S.A., Fitzgerald M. (2012). Myelin sheath decompaction, axon swelling, and functional loss during chronic secondary degeneration in rat optic nerve. Investig. Ophthalmol. Vis. Sci..

[35] Savigni D.L., O’Hare Doig R.L., Szymanski C.R., Bartlett C.A., Lozić I., Smith N.M., Fitzgerald M. (2013). Three Ca2+ channel inhibitors in combination limit chronic secondary degeneration following neurotrauma. Neuropharmacology.

[36] Payne S.C., Bartlett C.A., Savigni D.L., Harvey A.R., Dunlop S.A., Fitzgerald M. (2013). Early proliferation does not prevent the loss of oligodendrocyte progenitor cells during the chronic phase of secondary degeneration in a CNS white matter tract. PLoS ONE.

[37] Szymanski C.R., Chiha W., Morellini N., Cummins N., Bartlett C.A., O’Hare Doig R.L., Savigni D.L., Payne S.C., Harvey A.R., Dunlop S.A. (2013). Paranode Abnormalities and Oxidative Stress in Optic Nerve Vulnerable to Secondary Degeneration: Modulation by 670 nm Light Treatment. PLoS ONE.

[38] Chiha W., Bartlett C.A., Petratos S., Fitzgerald M., Harvey A.R. (2020). Intravitreal application of AAV-BDNF or mutant AAV-CRMP2 protects retinal ganglion cells and stabilizes axons and myelin after partial optic nerve injury. Exp. Neurol..

[39] Fitzgerald M., Bartlett C.A., Harvey A.R., Dunlop S.A. (2010). Early events of secondary degeneration after partial optic nerve transection: An immunohistochemical study. J. Neurotrauma.

[40] Donovan V., Kim C., Anugerah A.K., Coats J.S., Oyoyo U., Pardo A.C., Obenaus A. (2014). Repeated mild traumatic brain injury results in long-term white-matter disruption. J. Cereb. Blood Flow Metab..

[41] Chen H.S.-M., Holmes N., Liu J., Tetzlaff W., Kozlowski P. (2017). Validating myelin water imaging with transmission electron microscopy in a rat spinal cord injury model. NeuroImage.

[42] Nashmi R., Fehlings M.G. (2001). Changes in axonal physiology and morphology after chronic compressive injury of the rat thoracic spinal cord. Neuroscience.

[43] Anthes D.L., Theriault E., Tator C.H. (1995). Characterization of axonal ultrastructural pathology following experimental spinal cord compression injury. Brain Res..

[44] Lebel C., Walker L., Leemans A., Phillips L., Beaulieu C. (2008). Microstructural maturation of the human brain from childhood to adulthood. NeuroImage.

[45] Seidl A.H. (2014). Regulation of conduction time along axons. Neuroscience.

[46] Gutiérrez R., Boison D., Heinemann U., Stoffel W. (1995). Decompaction of CNS myelin leads to a reduction of the conduction velocity of action potentials in optic nerve. Neurosci. Lett..

[47] Boiko T., Rasband M.N., Levinson S.R., Caldwell J.H., Mandel G., Trimmer J.S., Matthews G. (2001). Compact Myelin Dictates the Differential Targeting of Two Sodium Channel Isoforms in the Same Axon. Neuron.

[48] Balraj A., Clarkson-Paredes C., Pajoohesh-Ganji A., Kay M.W., Mendelowitz D., Miller R.H. (2022). Refinement of axonal conduction and myelination in the mouse optic nerve indicate an extended period of postnatal developmental plasticity. Dev. Neurobiol..

[49] Ferrer E., Whitaker K.J., Steele J.S., Green C.T., Wendelken C., Bunge S.A. (2013). White matter maturation supports the development of reasoning ability through its influence on processing speed. Dev. Sci..

[50] Turken U., Whitfield-Gabrieli S., Bammer R., Baldo J.V., Dronkers N.F., Gabrieli J.D.E. (2008). Cognitive processing speed and the structure of white matter pathways: Convergent evidence from normal variation and lesion studies. NeuroImage.

[51] Sánchez I., Hassinger L., Paskevich P.A., Shine H.D., Nixon R.A. (1996). Oligodendroglia regulate the regional expansion of axon caliber and local accumulation of neurofilaments during development independently of myelin formation. J. Neurosci..

[52] Geeraert B.L., Lebel R.M., Mah A.C., Deoni S.C., Alsop D.C., Varma G., Lebel C. (2018). A comparison of inhomogeneous magnetization transfer, myelin volume fraction, and diffusion tensor imaging measures in healthy children. NeuroImage.

[53] Hüppi P.S., Dubois J. (2006). Diffusion tensor imaging of brain development. Semin. Fetal Neonatal Med..

[54] Yoshida S., Oishi K., Faria A.V., Mori S. (2013). Diffusion tensor imaging of normal brain development. Pediatr. Radiol..

[55] Wells J., Kilburn M.R., Shaw J.A., Bartlett C.A., Harvey A.R., Dunlop S.A., Fitzgerald M. (2012). Early in vivo changes in calcium ions, oxidative stress markers, and ion channel immunoreactivity following partial injury to the optic nerve. J. Neurosci. Res..

[56] Toomey L.M., Bartlett C.A., Majimbi M., Gopalasingam G., Rodger J., Fitzgerald M. (2019). Comparison of ion channel inhibitor combinations for limiting secondary degeneration following partial optic nerve transection. Exp. Brain Res..

[57] Toomey L.M., Bartlett C.A., Gavriel N., McGonigle T., Majimbi M., Gopalasingam G., Rodger J., Fitzgerald M. (2019). Comparing modes of delivery of a combination of ion channel inhibitors for limiting secondary degeneration following partial optic nerve transection. Sci. Rep..

[58] Cummins N., Bartlett C.A., Archer M., Bartlett E., Hemmi J.M., Harvey A.R., Dunlop S.A., Fitzgerald M. (2013). Changes to mitochondrial ultrastructure in optic nerve vulnerable to secondary degeneration in vivo are limited by irradiation at 670 nm. BMC Neurosci..

[59] Chiang A.C.A., Seua A.V., Singhmar P., Arroyo L.D., Mahalingam R., Hu J., Kavelaars A., Heijnen C.J. (2020). Bexarotene normalizes chemotherapy-induced myelin decompaction and reverses cognitive and sensorimotor deficits in mice. Acta Neuropathol. Commun..

[60] Chu P.H., Li H.-Y., Chin M.-P., So K.-f., Chan H.H. (2013). Effect of lycium barbarum (wolfberry) polysaccharides on preserving retinal function after partial optic nerve transection. PLoS ONE.

[61] Levkovitch-Verbin H., Spierer O., Vander S., Dardik R. (2011). Similarities and differences between primary and secondary degeneration of the optic nerve and the effect of minocycline. Graefe’s Arch. Clin. Exp. Ophthalmol..

[62] Ghasemi A., Jeddi S., Kashfi K. (2021). The laboratory rat: Age and body weight matter. EXCLI J..

[63] Reeves T.M., Smith T.L., Williamson J.C., Phillips L.L. (2012). Unmyelinated axons show selective rostrocaudal pathology in the corpus callosum after traumatic brain injury. J. Neuropathol. Exp. Neurol..

[64] Giacci M.K., Bartlett C.A., Huynh M., Kilburn M.R., Dunlop S.A., Fitzgerald M. (2018). Three dimensional electron microscopy reveals changing axonal and myelin morphology along normal and partially injured optic nerves. Sci. Rep..

[65] Kelley B.J., Farkas O., Lifshitz J., Povlishock J.T. (2006). Traumatic axonal injury in the perisomatic domain triggers ultrarapid secondary axotomy and Wallerian degeneration. Exp. Neurol..

[66] Saggu S.K., Chotaliya H.P., Blumbergs P.C., Casson R.J. (2010). Wallerian-like axonal degeneration in the optic nerve after excitotoxic retinal insult: An ultrastructural study. BMC Neurosci..

[67] Fehily B., Bartlett C.A., Lydiard S., Archer M., Milbourn H., Majimbi M., Hemmi J.M., Dunlop S.A., Yates N.J., Fitzgerald M. (2019). Differential responses to increasing numbers of mild traumatic brain injury in a rodent closed-head injury model. J. Neurochem..

[68] Wei J., Carroll R.J., Harden K.K., Wu G. (2012). Comparisons of treatment means when factors do not interact in two-factorial studies. Amino Acids.

