# Supplementary material for: Secondary Degeneration Impairs Myelin Ultrastructural Development in Adulthood following Adolescent Neurotrauma in the Rat Optic Nerve
Source: Int J Mol Sci. 2023 Feb 7;24(4):3343. doi: 10.3390/ijms24043343 (PMC9966883; doi:10.3390/ijms24043343)
Supplement: Supplementary file 1 [file ijms-24-03343-s001.zip › ijms-2134449-supplementary.pdf]

Table S1. All 2-way ANOVA Results.

**Myelin Classifications**				
**Measurement**	**Age (Main effect)**	**Injury (Main Effect)**	**Interaction Effect**	**Figure**
Axons with Compact Myelin (%)	$F_{(1,16)} = 27.36$, $p < 0.0001$	$F_{(1,16)} = 2.388$, $p = 0.1418$	$F_{(1,16)} = 3.912$, $p = 0.0654$	3a
Axons with moderately decompacted myelin (%)	$F_{(1,16)} = 16.20$, $p = 0.0010$	$(F_{(1,16)} = 0.4280$, $p = 0.5223$	$F_{(1,16)} = 0.3559$, $p = 0.5592$	3b
Axons with severely decompacted myelin (%)	$F_{(1,16)} = 66.91$, $p < 0.0001$	$F_{(1,16)} = 4.100$, $p = 0.0599$	$F_{(1,16)} = 10.80$, $p = 0.0046$	3c
Unmyelinated axons (%)	$F_{(1,16)} = 5.871$, $p = 0.0276$	$F_{(1,16)} = 0.4292$, $p = 0.5217$	$F_{(1,16)} = 0.1694$, $p = 0.6861$	3d
**Morphology of Axons with Compact Myelin**				
**Measurement**	**Age (Main effect)**	**Injury (Main Effect)**	**Interaction Effect**	**Figure**
Myelin Thickness (μm)	$F_{(1,16)} = 21.05$, $p = 0.0003$	$F_{(1,16)} = 0.001438$, $p = 0.9702$	$F_{(1,16)} = 8.525$, $p = 0.0100$	4a
Axon Diameter (μm)	$F_{(1,16)} = 1.627$, $p = 0.2204$	$F_{(1,16)} = 0.1320$, $p = 0.7211$	$F_{(1,16)} = 0.001$, $p = 0.9904$	4b
Fibre Diameter (μm)	$F_{(1,16)} = 2.793$, $p = 0.1141$	$F_{(1,16)} = 0.04561$, $p = 0.8336$	$F_{(1,16)} = 0.1746$, $p = 0.6816$	4c
G Ratio	$F_{(1,16)} = 0.3602$, $p = 0.5568$	$F_{(1,16)} = 0.07953$, $p = 0.7815$	$F_{(1,16)} = 1.130$, $p = 0.3036$	4d
**Diameter of Axons with Decompacted Myelin**				
**Measurement**	**Age (Main effect)**	**Injury (Main Effect)**	**Interaction Effect**	**Figure**
Axons with Moderately Decompacted Myelin (μm)	$F_{(1,16)} = 6.096$, $p = 0.0252$	$F_{(1,16)} = 2.703$, $p = 0.1196$	$F_{(1,16)} = 0.07367$, $p = 0.7895$	4e
Axons with Severely Decompacted Myelin (μm)	$F_{(1,16)} = 1.807$, $p = 0.1977$	$F_{(1,16)} = 2.191$, $p = 0.1582$	$F_{(1,16)} = 0.7760$; $p = 0.3914$	4f
Axons with Completely Decompacted Myelin (μm)	$F_{(1,14)} = 0.1275$, $p = 0.7264$	$F_{(1,14)} = 0.5569$, $p = 0.4679$	$F_{(1,14)} = 2.709$, $p = 0.1220$	4g
Unmyelinated Axons (μm)	$F_{(1,13)} = 0.02401$, $p = 0.8792$	$F_{(1,13)} = 4.47$, $p = 0.0542$	$F_{(1,13)} = 0.1578$, $p = 0.6977$	4h
**Population Analyses**				
Measurement	**Age (Main effect)**	**Injury (Main Effect)**	**Interaction Effect**	**Figure**
Median Diameter of Axons with Compact Myelin	$F_{(1,16)} = 1.059$, $p = 0.3186$	$F_{(1,16)} = 0.1351$, $p = 0.7180$	$F_{(1,16)} = 0.7784$, $p = 0.3907$	5e
Median Diameter of Axons with Decompacted Myelin	$F_{(1,16)} = 1.247$, $p = 0.2807$	$F_{(1,16)} = 1.086$, $p = 0.3129$	$F_{(1,16)} = 0.0886$, $p = 0.7697$	5f
